# Distribution of gamma radiation dose rate related with natural radionuclides in all of Vietnam and radiological risk assessment of the built-up environment

**DOI:** 10.1038/s41598-020-69003-0

**Published:** 2020-07-24

**Authors:** Kazumasa Inoue, Masahiro Fukushi, Tan Van Le, Hiroshi Tsuruoka, Shogo Kasahara, Veerasamy Nimelan

**Affiliations:** 1 Department of Radiological Sciences, Graduate School of Human Health Sciences, Tokyo Metropolitan University, Tokyo, 116-8551 Japan; 2 Department of Radiology, Cho Ray Hospital, Ho Chi Minh City, 72713 Vietnam; 3 Department of Radiological Sciences, Tsukuba International University, Ibaraki, 300-0051 Japan

**Keywords:** Environmental sciences, Natural hazards

## Abstract

A built-up environment utilizes building materials containing natural radionuclides that will change radiological risks. While radiological risks have been estimated from the activity concentrations of natural radionuclides in soil, it is important to evaluate the changes of these risks for the built-up environment using these building materials. Based on the direct measurements of absorbed dose rate in air and calculation of absorbed dose rate in air from activity concentrations in soil for all of Vietnam which has undergone significant economic growth in recent decades, the changes of absorbed dose rate in air and radiological risks before and after construction of many artificial structures were investigated. The results showed that the absorbed dose rates in air were clearly changed by the urbanization, and the difference ratio for all of Vietnam ranged from 0.5 to 2.1, meaning that the artificial structures have been acting as shielding materials to terrestrial gamma-rays or radiation sources. However, changes in annual effective dose in the built-up environment were small, and there was no new radiation risk from the built-up environment for Vietnam.

## Introduction

The exposure of human beings to ionizing radiation from natural sources is related to high-energy cosmic ray particles, human activities, construction materials and geological structures; and both external and internal exposures to humans arise from these sources^1^. Gamma-rays emitted from natural sources are largely due to ^238^U series and ^232^Th series elements and their decay products, as well as ^40^K which exist at trace levels in the earth’s crust^2–5^. The measurement of these natural sources allows determination of the background baseline of natural radioactivity^6^ and estimation of the radiation hazard risks to humans^7,8^. It is well known that construction materials of concrete, brick, sandstone, granite and other natural stones contain these natural sources. Depending on their amounts, these sources may cause changes in radiation level of the built-up environment which means the environment made by the natural environment, artificial structures (i.e., roads, buildings and bridges) and human activities; as a consequence, the radiological risks from such natural radioactivity will be changed^9,10^. It can be assumed that such changes may be occurring as a result of remarkable economic growth accompanied with newly constructed artificial structures.

In Vietnam, the real GDP (gross domestic product) growth rate in 2018 was 7.1%, and that was the highest growth rate in the past 10 years. Therefore, an impact can be assumed on the radiation level from natural radionuclides contained within the materials of newly constructed artificial structures, especially roads and high-rise buildings, resulting in changed radiation level and hazard risks related with them. The contents of natural radionuclides in the soil are very important because these radionuclides contribute significantly to the collective dose of the general population^11^. Some researchers have measured activity concentrations of natural radionuclides in soil in Vietnam^12–14^. The most recent observations^13^ for all of Vietnam showed that the average concentrations of ^238^U, ^232^Th and ^40^K were 43 ± 18 Bq kg^−1^, 60 ± 20 Bq kg^−1^ and 442 ± 230 Bq kg^−1^, respectively, which are 1.2, 2.0 and 1.0 times higher than the worldwide averages^1^. On the other hand, the total annual effective dose^6^, radium equivalent activity and external hazard index were estimated to be 0.54 mSv year^−1^, 160 Bq kg^−1^ and 0.43, respectively; as a consequence, it was concluded that exposure to Vietnamese soil as a building material (primarily as brick) was not an increased hazard for the human population^14^. A similar assessment was carried out in Sri Lanka^15^ and Ireland^16^, and similar results were reported. On the other hand, there are areas which have discouraged use of building materials from high natural background radiation areas and one example is Iran^10^. However, there has been no investigation to assess changes in radiation level of the built-up environment which is made by artificial structures such as roads, buildings and bridges constructed from building materials. The presence of building materials containing natural radionuclides in urban areas can be expected to cause either an increased radiation level or a decreased radiation level^17^. The former results from the presence of building materials which contain high concentrations of natural radioactivity sources^18,19^. For example, bricks contain such radioactive materials as uranium and thorium. While the levels of radiation present in bricks are low, they are higher than in some other building materials used for homes, such as wood^20^. The latter results from a shielding effect toward terrestrial gamma-rays and it depends on the amount and distribution of the building materials and the background level^21^. The investigation of radiation level in relation to changes of the built-up environment in urban areas can provide a better understanding for environmental impact assessment of urban development. A way to investigate this impact is offered by the direct measurement of absorbed dose rates^6^ in air 1 m above the ground surface from terrestrial gamma-rays and artificial structures, followed by a comparison of the estimated absorbed dose rates in air 1 m above the ground surface based on activity concentrations of ^238^U, ^232^Th and ^40^K in soil samples (i.e., from only terrestrial gamma-rays).

In this study, the measurements of absorbed dose rates in air 1 m above the ground surface were carried out for all of Vietnam. From them the detailed dose distribution map of the background related with natural radionuclides contained in soil and building materials was obtained; this map can be used for investigating external effective doses^6,18^. Additionally, external effective dose and the external hazard index were calculated using data obtained from direct measurements of absorbed dose rate in air and from activity concentrations in soil samples collected at 426 locations. Finally, the impact on the radiological hazard index of artificial structures utilizing low-level radiation building materials in urban areas was estimated. This study is the first large-scale attempt in Vietnam to assess changes of radiological risk on the built-up environment in relation to economic growth.

## Results

### Absorbed dose rates in air and activity concentrations of ^238^U, ^232^Th and ^40^K

**Figure 1:**
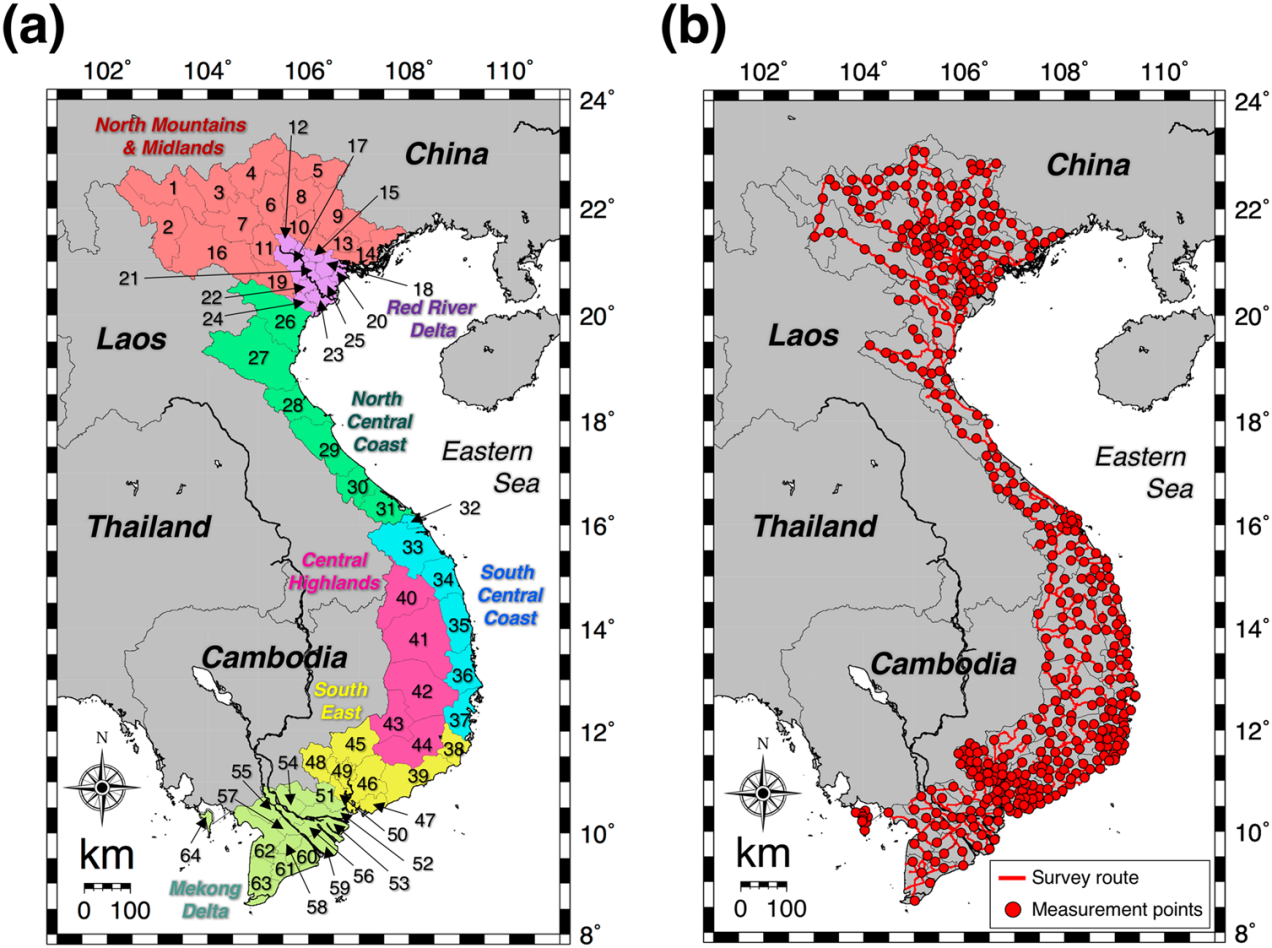
(**a**) Map showing Vietnam administrative divisions consisting of 53 provinces and five cities. While Phu Quoc Island (#64) is part of Kien Giang Province (#62), it is given here as a separate listing due to its island character and size. (**b**) The survey routes (red lines) for measuring the count rates. Total distance traveled was 28,201 km. The red circles represent points for fixed-point measurements and collection of soil samples (*n* = 462). These maps drawn using the Generic Mapping Tools, version 4.5.18 (https://gmt.soest.hawaii.edu/gmt4/).

**Figure 2:**
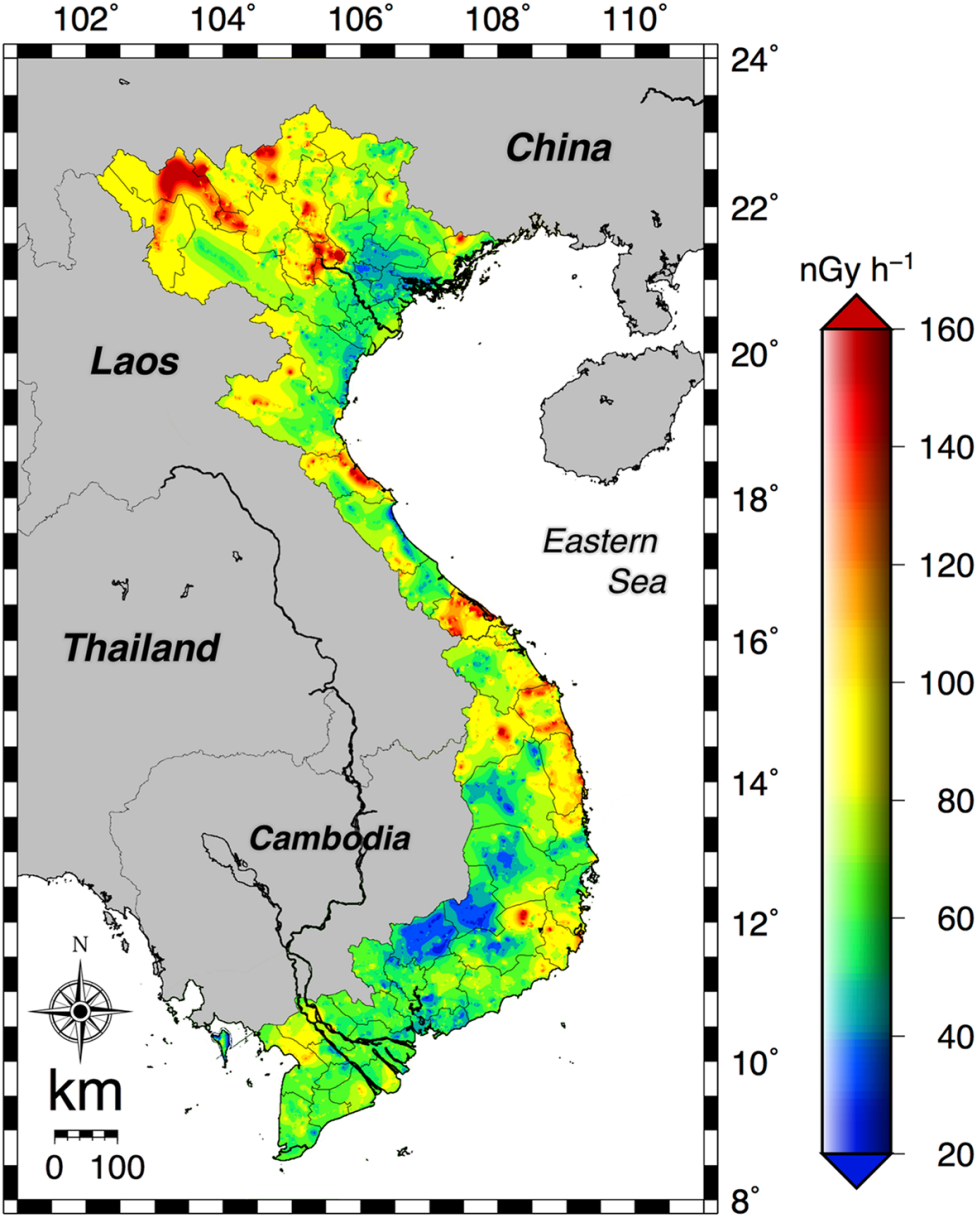
Distribution map of absorbed dose rates in air 1 m above the ground surface measured in the car-borne survey. This map was drawn using 80,516 data. This map drawn using the Generic Mapping Tools, version 4.5.18 (https://gmt.soest.hawaii.edu/gmt4/).

**Figure 3:**
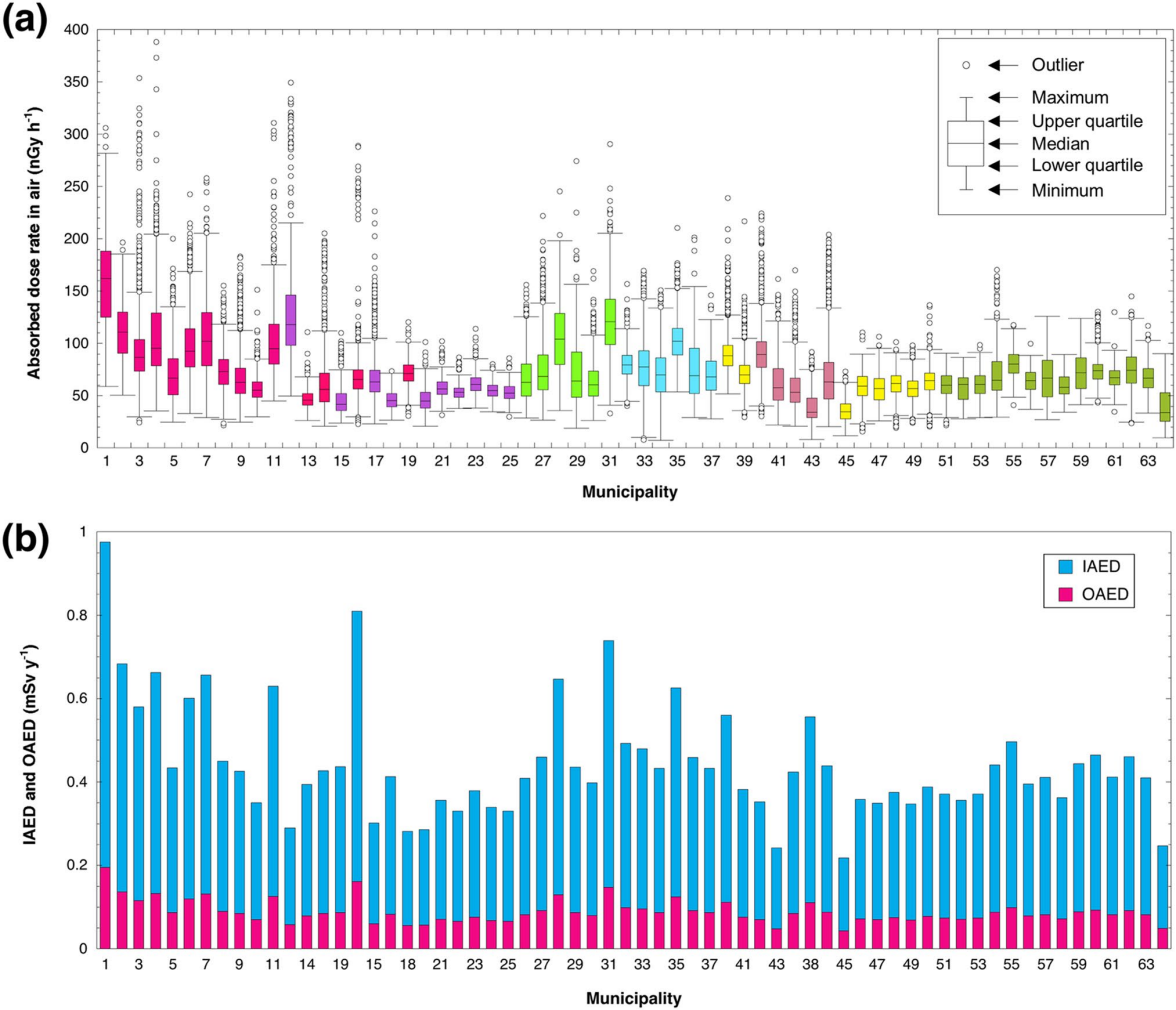
(**a**) Box-and-whisker diagram of absorbed dose rates in air in all municipalities in Vietnam based on the measurements in the car-borne survey (*n* = 80,515). (**b**) Annual indoor (IAED) and outdoor (OAED) annual effective doses in all municipalities.

The absorbed dose rates in air 1 m above the grand surface were measured by the car-borne survey technique for 58 provinces and five cities in Vietnam (Fig. 1a). This survey was carried out by positioning a dosimeter inside the car and driving on asphalt pavements (Fig. 1b). The measured dose rates inside the car were corrected by multiplying with shielding factors in order to represent unshielded external dose rates above a bare surface. The detailed dose rate distribution map for all of Vietnam is shown in Fig. 2. A heterogeneous distribution of absorbed dose rate in air was seen (*n* = 80,516). Dose rates of over 160 nGy h^−1^ were found in the north mountain area (#1 in Fig. 1a) whereas lower dose rates of under 35 nGy h^−1^ were found in the southeast area (#43, #45 and #64 in Fig. 1a). The average (range) of absorbed dose rate in air for all of Vietnam was estimated to be 75 ± 32 nGy h^−1^ (8–463 nGy h^−1^). Those values for northern Vietnam (#1–31 in Fig. 1a; *n* = 33,605) and southern Vietnam (#32–64 in Fig. 1a; *n* = 46,911) were 84 ± 39 nGy h^−1^ (19–463 nGy h^−1^) and 68 ± 24 nGy h^−1^ (8–239 nGy h^−1^), respectively; hence, the dose rates measured in northern Vietnam tended to be higher than those in southern Vietnam. The detailed dose rates in each municipality are shown in Fig. 3a. The maximum combined relative standard uncertainty involved in absorbed dose rate in air was 11.5%^22,23^. The highest dose rate was observed in Lai Chau Province (#1 in Fig. 1a), and the average (range) absorbed dose rate in air was 159 ± 42 nGy h^−1^ (59–306 nGy h^−1^), whereas the lowest value was observed in Binh Phuoc Province (#45 in Fig. 1a), and the average (range) dose rate was 36 ± 10 nGy h^−1^ (12–73 nGy h^−1^). Based on those calculated absorbed dose rates in air, the annual effective doses of all of Vietnam (*n* = 80,516) were 0.37 mSv year^−1^ (0.04–2.27 mSv year^−1^) for the annual indoor effective dose (IAED) and 0.09 mSv year^−1^ (0.01–0.57 mSv year^−1^) for the annual outdoor effective dose (OAED). The average of the total annual effective dose (IAED + OAED) was 0.46 mSv year^−1^ (0.05–2.84 mSv year^−1^) and that was similar to the worldwide average (0.48 mSv year^−1^)^21^. The detailed estimated annual effective dose for each province is summarized in Fig. 3b.

**Figure 4:**
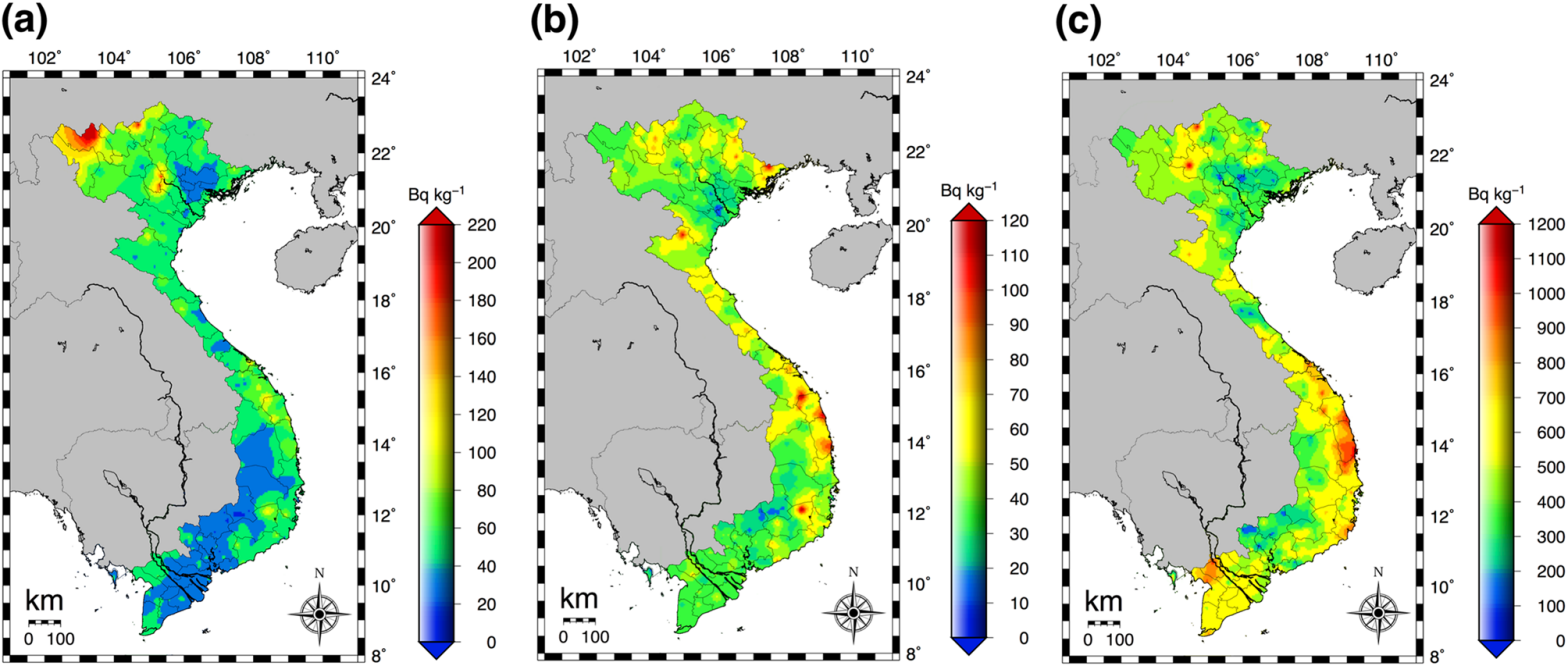
Activity concentrations of the natural radionuclides obtained from absorbed dose rates in air and soil in all municipalities in Vietnam (*n* = 462): (**a**) ^238^U, (**b**) ^232^Th and (**c**) ^40^K. These maps drawn using the Generic Mapping Tools, version 4.5.18 (https://gmt.soest.hawaii.edu/gmt4/).

The distribution maps of ^238^U, ^232^Th and ^40^K in Bq kg^−1^ for all of Vietnam are shown in Fig. 4. The activity concentrations of each natural radionuclide were calculated from direct measurements of the gamma-ray pulse height distributions 1 m above the ground surface by fixed-point observations at 462 locations (red circles in Fig. 1b). The maximum combined relative standard uncertainty involved in activity concentration in air was 9.6%^22,23^. Higher activity concentrations of ^238^U over 170 Bq kg^−1^ were observed in the north mountain area (#1 in Fig. 1a) whereas lower activity concentrations under 35 Bq kg^−1^ were observed in Bac Giang Province (#13 in Fig. 1a) and southern Vietnam excluding the south central coast area. Contrary to this tendency, higher activity concentrations of ^232^Th over 70 Bq kg^−1^ and of ^40^K over 700 Bq kg^−1^ were observed in the south central coast area (#32, #34 and #35 in Fig. 1a). The detailed activity concentrations of ^238^U, ^232^Th and ^40^K in all of Vietnam and in each municipality can be found in Table 1 and Supplementary Table S1, respectively. The calculated average (range) of absorbed dose rates in air obtained from fixed-point measurements (*n* = 462) was 71 ± 28 nGy h^−1^ (20–217 nGy h^−1^), and the detailed dose rates for each municipality are shown in Table 2. Based on those analyzed activity concentrations, the annual effective doses of all of Vietnam (*n* = 462) were 0.38 mSv year^−1^ (0.03–1.05 mSv year^−1^) for the IAED and 0.09 mSv year^−1^ (0.01–0.26 mSv year^−1^) for the OAED as shown in Table 3. The average of the total annual effective dose (IAED + OAED) was 0.47 mSv year^−1^ (0.04–1.31 mSv year^−1^) and that was slightly higher value compared to calculated values from direct measurement. Additionally, that was similar to the worldwide average (0.48 mSv year^−1^)^21^.

**Table 1:** Activity concentrations of natural radionuclides from direct measurements and soil concentrations.

Measurement method Measurement area	*n*	^238^U (Bq kg^−1^)		^232^Th (Bq kg^−1^)		^40^K (Bq kg^−1^)		Ra_eq_ (Bq kg^−1^)		H_ex_	
		Average	Range	Average	Range	Average	Range	Average	Range	Average	Range
**Direct measurement**											
All of Vietnam	462	50 ± 32	12–395	43 ± 20	9–157	486 ± 228	30–1,319	149 ± 62	44–464	0.4 ± 0.2	0.1–1.3
Northern Vietnam^a^	178	60 ± 43	20–395	44 ± 20	9–130	415 ± 208	30–1,319	155 ± 66	61–464	0.4 ± 0.2	0.2–1.3
Southern Vietnam^b^	284	44 ± 20	12–155	43 ± 21	11–157	531 ± 230	56–1,179	146 ± 60	44–422	0.4 ± 0.2	0.1–1.1
**Soil concentration**											
All of Vietnam	462	35 ± 22	0–160	57 ± 33	0–173	551 ± 342	4–1910	159 ± 82	11–435	0.4 ± 0.2	0.0–1.2
Northern Vietnam^a^	178	39 ± 25	6–149	63 ± 33	2–168	551 ± 259	23–1,320	171 ± 78	19–398	0.5 ± 0.2	0.1–1.1
Southern Vietnam^b^	284	32 ± 19	0–160	54 ± 33	0–173	551 ± 389	4–1910	151 ± 84	11–435	0.4 ± 0.2	0.0–1.2

^a^Northern Vietnam refers to #1–#31 in Fig. 1a.

^b^Southern Vietnam refers to #32–#64 in Fig. 1a.

**Table 2:** Calculated absorbed dose rate in air from direct measurements and soil concentration.

No.^a^	Municipality	*n*	Absorbed dose rate in air (nGy h^−1^)				Ratio of difference
			Direct measurement		Soil concentration		
			Mean ± SD	Range	Mean ± SD	Range	
1	Lai Chau	6	116 ± 37	87–186	116 ± 35	63–159	1.0
2	Dien Bien	3	88 ± 24	63–110	121 ± 6	115–126	0.7
3	Lao Cai	6	81 ± 27	47–110	110 ± 39	49–155	0.7
4	Ha Giang	10	92 ± 51	44–217	118 ± 45	51–184	0.8
5	Cao Bang	6	60 ± 31	37–119	56 ± 17	27–81	1.1
6	Tuyen Quang	7	78 ± 22	49–120	107 ± 44	53–190	0.7
7	Yen Bai	5	78 ± 38	30–117	94 ± 51	16–152	0.8
8	Bac Kan	7	72 ± 17	47–100	89 ± 24	44–122	0.8
9	Lang Son	10	83 ± 29	43–131	97 ± 30	39–131	0.9
10	Thai Nguyen	6	56 ± 23	38–99	52 ± 32	27–116	1.1
11	Phu Tho	12	78 ± 19	52–113	84 ± 25	49–136	0.9
12	Vinh Phuc	3	89 ± 8	84–98	124 ± 18	108–144	0.7
13	Bac Giang	8	47 ± 10	31–64	49 ± 16	29–70	1.0
14	Quang Ninh	8	84 ± 32	44–144	76 ± 34	39–139	1.1
15	Bac Ninh	2	42 ± 1	41–43	46 ± 1	45–47	0.9
16	Son La	6	60 ± 12	43–75	93 ± 21	78–133	0.7
17	Ha Noi	3	48 ± 8	42–57	79 ± 21	55–95	0.6
18	Hai Duong	3	43 ± 4	39–46	53 ± 13	44–62	0.8
19	Hoa Binh	5	67 ± 17	48–93	75 ± 17	49–95	0.9
20	Hai Phong	2	60 ± 13	50–69	71 ± 2	69–72	0.8
21	Hung Yen	3	52 ± 10	41–61	60 ± 30	25–82	0.9
22	Ha Nam	3	43 ± 8	37–52	73 ± 29	48–104	0.6
23	Thai Binh	3	57 ± 2	56–59	84 ± 9	74–92	0.7
24	Ninh Binh	3	52 ± 4	48–56	86 ± 39	44–120	0.6
25	Nam Dinh	3	45 ± 16	32–63	52 ± 21	33–75	0.9
26	Thanh Hoa	6	69 ± 32	32–104	62 ± 21	38–98	1.1
27	Nghe An	14	78 ± 28	48–154	80 ± 36	30–144	1.0
28	Ha Tinh	4	80 ± 20	57–106	58 ± 18	39–85	1.4
29	Quang Binh	9	73 ± 23	37–98	78 ± 43	26–162	0.9
30	Quang Tri	7	76 ± 24	45–115	78 ± 39	9–116	1.0
31	Thua Thien-Hue	5	86 ± 28	62–131	94 ± 56	16–181	0.9
32	Da Nang	8	101 ± 9	89–119	83 ± 49	30–157	1.2
33	Quang Nam	14	84 ± 22	48–114	81 ± 28	45–140	1.0
34	Quang Ngai	11	116 ± 40	64–175	91 ± 35	50–163	1.3
35	Binh Dinh	11	117 ± 23	85–151	117 ± 58	41–214	1.0
36	Phu Yen	8	81 ± 22	60–114	109 ± 43	65–178	0.7
37	Khanh Hoa	19	72 ± 17	26–98	98 ± 32	49–163	0.7
38	Ninh Thuan	20	83 ± 17	56–121	93 ± 47	18–184	0.9
39	Binh Thuan	18	65 ± 19	28–107	71 ± 32	16–124	0.9
40	Kon Tum	7	79 ± 21	44–101	106 ± 39	63–146	0.7
41	Gia Lai	11	61 ± 23	33–104	98 ± 49	51–205	0.6
42	Dac Lac	11	61 ± 18	38–94	65 ± 47	20–148	0.9
43	Dac Nong	9	37 ± 17	22–73	48 ± 22	14–84	0.8
44	Lam Dong	14	80 ± 39	41–193	80 ± 36	32–151	1.0
45	Binh Phuoc	4	34 ± 19	20–62	66 ± 51	20–136	0.5
46	Dong Nai	15	56 ± 14	27–77	61 ± 17	25–85	0.5
47	Ba Ria-Vung Tau	7	48 ± 11	36–69	44 ± 20	17–73	1.1
48	Tay Ninh	20	50 ± 14	29–80	47 ± 14	14–71	1.1
49	Binh Duong	11	52 ± 10	36–68	56 ± 28	27–104	0.9
50	Ho Chi Minh	11	52 ± 11	29–74	55 ± 17	23–78	0.9
51	Long An	4	75 ± 10	61–85	85 ± 29	59–136	0.9
52	Tien Giang	5	54 ± 9	42–66	49 ± 9	43–59	1.1
53	Ben Tre	4	59 ± 8	51–66	62 ± 7	56–72	0.9
54	Dong Thap	7	61 ± 12	49–80	76 ± 47	43–156	0.8
55	An Giang	3	93 ± 10	81–99	61 ± 23	40–84	1.5
56	Vinh Long	3	59 ± 12	49–73	119 ± 42	73–156	0.5
57	Can Tho	2	48 ± 22	32–63	106 ± 67	58–153	0.5
58	Hau Giang	1	71	ー	85	ー	0.8
59	Tra Vinh	2	54 ± 3	52–56	80 ± 21	56–94	0.7
60	Soc Trang	3	67 ± 13	54–79	75 ± 42	45–123	0.9
61	Bac Lieu	2	74 ± 1	73–74	52 ± 8	46–58	1.4
62	King Giang	7	79 ± 17	54–104	43 ± 44	5–171	1.8
63	Ca Mau	4	66 ± 12	52–81	92 ± 50	47–146	0.7
64	Phu Quoc Island	10	45 ± 22	26–95	22 ± 7	10–31	2.1
All of Vietnam		462	71 ± 28	20–217	77 ± 40	5–214	0.9
Northern Vietnam^b^		178	73 ± 29	30–217	83 ± 37	9–190	0.9
Southern Vietnam^c^		284	69 ± 28	20–193	74 ± 41	5–214	0.9

^a^The numbers refer to the designations in Fig. 1a.

^b^Northern Vietnam refers to #1–#31 in Fig. 1a.

^c^Southern Vietnam refers to #32–#64 in Fig. 1a.

**Table 3:** Calculated annual effective doses from direct measurements and soil concentrations.

Measurement method Measurement area	*n*	IAED (mSv year^−1^)		OAED (mSv year^−1^)		Total (IAED + OAED) (mSv year^−1^)	
		Average	Range	Average	Range	Average	Range
**Direct measurement**							
All of Vietnam	462	0.35	0.10–1.06	0.09	0.03–0.27	0.44	0.13–1.33
Northern Vietnam^a^	178	0.36	0.15–1.06	0.09	0.03–0.27	0.45	0.18–1.33
Southern Vietnam^b^	284	0.34	0.01–0.96	0.08	0.03–0.24	0.42	0.04–1.20
**Soil concentration**							
All of Vietnam	462	0.38	0.03–1.05	0.09	0.01–0.26	0.47	0.04–1.31
Northern Vietnam^a^	178	0.41	0.05–0.93	0.10	0.01–0.23	0.51	0.06–1.16
Southern Vietnam^b^	284	0.36	0.01–1.05	0.09	0.01–0.26	0.45	0.01–1.31

^a^Northern Vietnam refers to #1–#31 in Fig. 1a.

^b^Southern Vietnam refers to #32–#64 in Fig. 1a.

### Activity concentrations of ^238^U, ^232^Th, ^40^K and Ra_eq_ in soil samples

**Figure 5:**
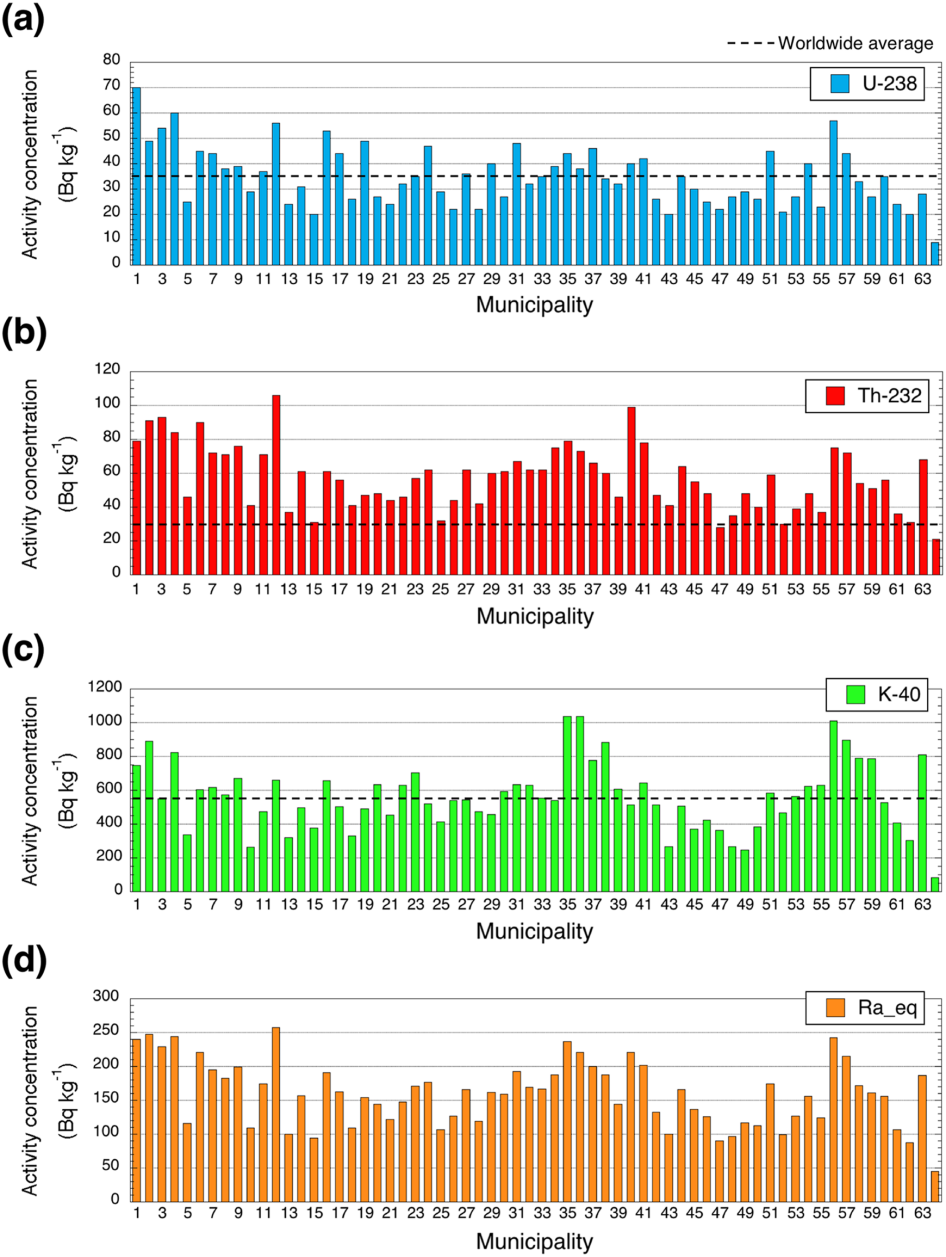
Activity concentrations for each municipality of (**a**) ^238^U, (**b**) ^232^Th, (**c**) ^40^K and (**d**) radium equivalent in soil.

The activity concentrations of ^238^U, ^232^Th, ^40^K and Ra_eq_ in soil (*n* = 462) for each municipality are shown in Fig. 5.

The soil samples were collected at the same locations as those for fixed-point measurements (Fig. 1b). The average activity concentrations of ^238^U, ^232^Th, ^40^K and Ra_eq_ for all of Vietnam were 35 ± 22 Bq kg^−1^, 57 ± 33 Bq kg^−1^, 551 ± 432 Bq kg^−1^, 159 ± 82 Bq kg^−1^, respectively; in other words, lower activity concentrations of ^238^U and higher activity concentrations of ^232^Th, ^40^K and Ra_eq_ were observed compared to data obtained from direct measurements (Table 1). The detailed activity concentrations of ^238^U, ^232^Th and ^40^K in soil in each municipality can be found in Supplementary Table S2. The maximum combined relative standard uncertainties involved in soil measurement were estimated to be 8.0% for ^238^U, 3.0% for ^232^Th and 2.3% for ^40^K^22,23^. The calculated average absorbed dose rates in air from soil concentrations of natural radionuclides are shown in Table 2. The average ratio of the difference (direct measurement/soil concentration in absorbed dose rate in air) for all of Vietnam was 0.9 (0.5–2.1); hence, a slightly lower dose rate was observed for the direct measurement.

### External radiological hazard index

**Figure 6:**
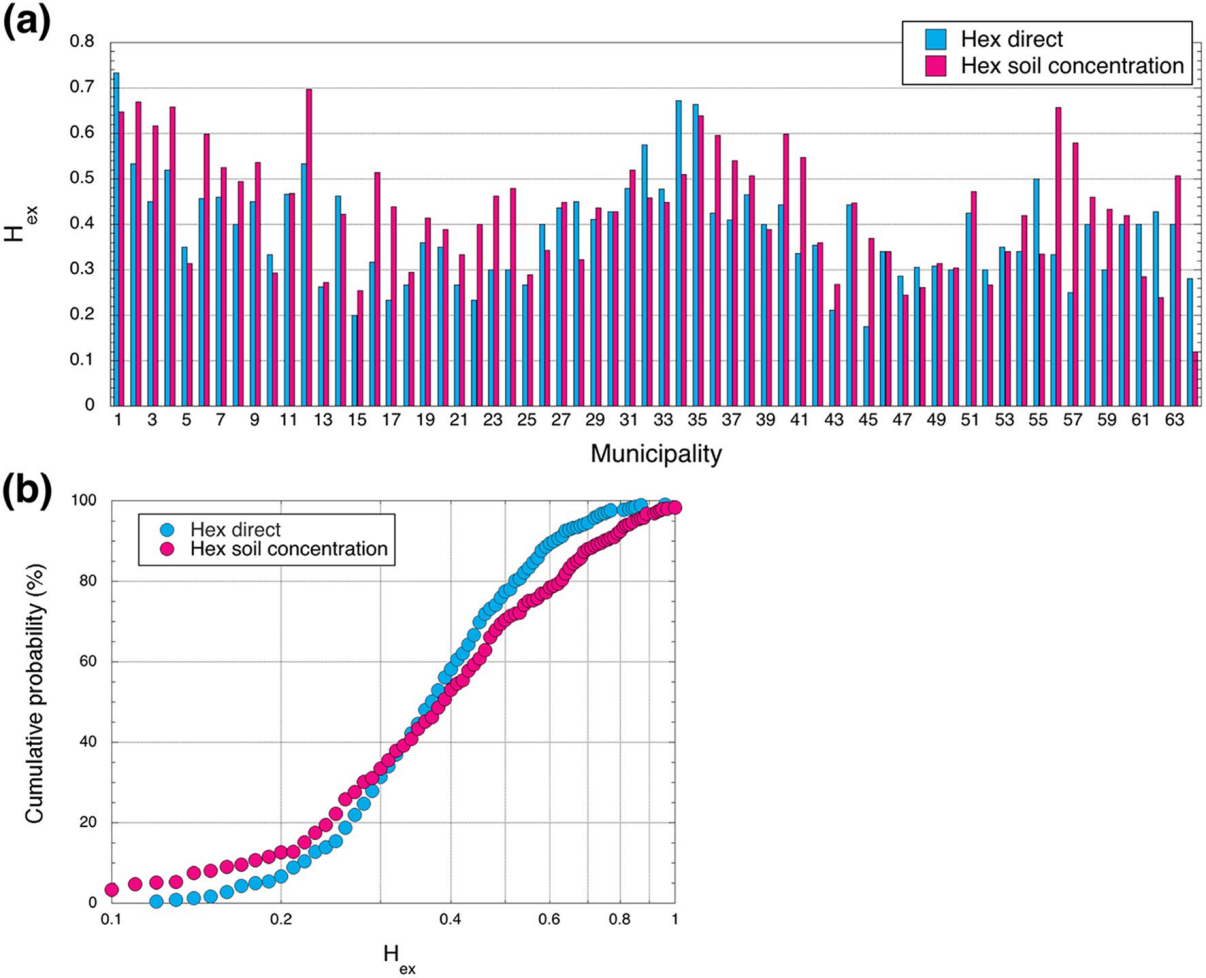
(**a**) External hazard index for each municipality in Vietnam. (**b**) Cumulative frequency distributions of external hazard index.

The calculated external hazard index values from direct measurements and from soil concentrations for each municipality are shown in Fig. 6a. The average H_ex_ calculated from direct measurements and from soil concentrations for all of Vietnam was 0.4 ± 0.2 with a range from 0.1 and 1.3 (Table 1) but there were large differences between those from direct measurements and soil concentrations depending on the municipality. In particular, Long An (#51), King Giang (#62) and Phu Quoc Island (#64) in the Mekong Delta had over 1.5 times higher H_ex_ values calculated from direct measurements compared to those from soil concentrations, whereas Son La (#16), Ha Noi (#17), Ha Nam (#22), Thai Binh (#23), Ninh Binh (#24), Gia Lai (#41), Binh Phuoc (#45), Vinh Long (#56) and Can Tho (#57) had over 1.5 times higher H_ex_ values for the same comparison. The detailed values for all of Vietnam can be found in Table 1. The cumulative frequency distributions of the external hazard index calculated from direct measurements and soil concentrations (*n* = 462) are shown in Fig. 6b. The cumulative frequency distributions showed clear differences in the H_ex_ between the measurement methods. About 65% of the H_ex_ values calculated from direct measurements using all data were lower compared to those from soil concentrations.

## Discussion

Determining natural radioactivity is of considerable importance for investigations of impacts on radiological risks due to artificial structures of urban development, radiation exposure in natural high background areas, and future exploitation of nuclear energy as well as investigations of the distribution of radionuclides in organs. According to the UNSCEAR report findings^21^, the average (range) of the outdoor absorbed dose rates in air in 25 countries was 59 nGy h^−1^ (18–93 nGy h^−1^). The results in the present study for all of Vietnam from direct measurement in the car-borne survey (75 ± 32 nGy h^−1^; *n* = 80,516) and fixed-point measurement (71 ± 28 nGy h^−1^; *n* = 462) were 1.2–1.3 times higher values compared to the worldwide average, but within the range.

**Figure 7:**
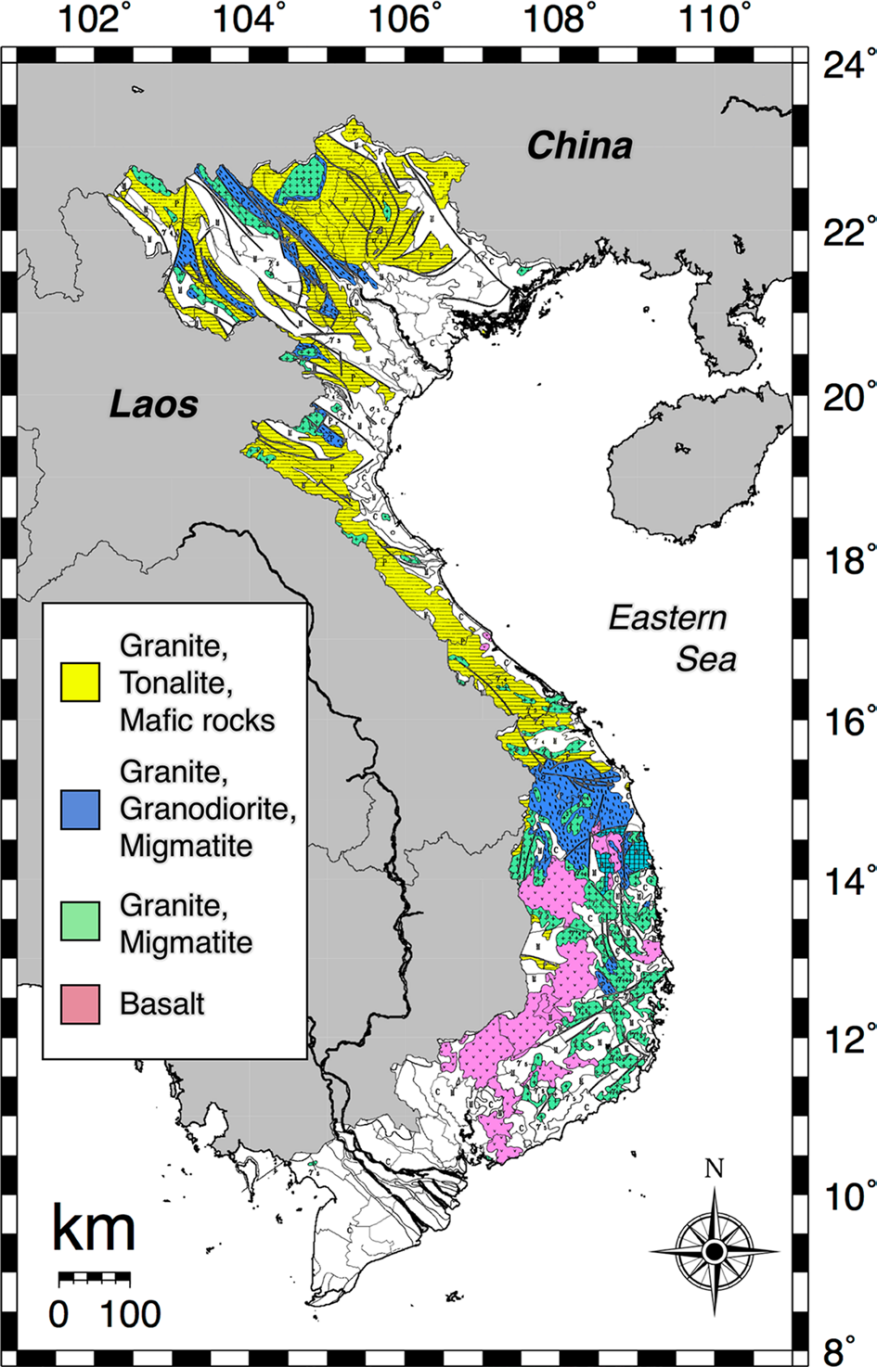
The geological map of Vietnam. These maps drawn using the Generic Mapping Tools, version 4.5.18 (https://gmt.soest.hawaii.edu/gmt4/).

The distribution map of absorbed dose rate in air had a heterogeneous distribution. It was found that 79% of all measured data exceeded the worldwide average, and 17% of all data exceeded 100 nGy h^−1^. The gamma radiation emitted by natural sources present in the earth’s crust, in building materials, air, water, food and the human body is largely due to primordial radionuclides, mainly ^40^K, ^238^U and ^232^Th series elements and their decay products. These primordial radionuclides have contributions of 35% for ^40^K, 25% for ^238^U and 40% for ^232^Th to the absorbed dose rate in air as gamma radiation^24^; consequently, the dose distribution depends on their activity concentrations^25^. Figure 7 presents a geographical map for Vietnam. The distribution of activity concentrations of these radionuclides mainly depended on basement geology as has been reported in the literature^26^. Southern Vietnam which showed a lower absorbed dose rate in air mainly has soil formed from basalt and ancient alluvial and poor nutrient sediments. Thus, the activity concentration of ^40^K is very dependent on dose rate because not much ^238^U and ^232^Th are contained in this rock and these sediments^14^. On the other hand, northern Vietnam has been formed by multiple types of rocks, including granite, tonalite, mafic rocks, granodiorite and migmatite. In those types, higher radiation levels are associated with igneous rocks such as granite which contains monazite (^232^Th), allanite (^232^Th) and zircon (^238^U and ^232^Th)^27^. The worldwide average activity concentrations of ^40^K, ^238^U and ^232^Th are 400 Bq kg^−1^, 35 Bq kg^−1^ and 30 Bq kg^−1^, respectively^21^, meaning that the average values of ^40^K and ^232^Th in the soil of all of Vietnam were 1.4 and 1.9 times higher than the worldwide average values.

Direct measurements of absorbed dose rate in air generally showed reasonable agreement with the calculated absorbed dose rate in air from soil concentration results. In previous studies, considerable discrepancies were observed for some countries such as Luxembourg, Sweden, Syria and Albania depending on the activity concentrations of natural radionuclides in soil; and the UNSCEAR report noted that a discrepancy of 30% or more indicated that a single survey should not be considered representative for a country^21^. Thus, the obtained result (ratio of the difference: 0.9) in the present study (Table 2) could be taken as representative for Vietnam.

For 65% of all direct measurement data (*n* = 462), a lower value (ratio of difference < 1.0) was obtained compared to that from soil concentrations. The absorbed dose rates in air in the built-up environment are significantly affected by artificial structures such as surface and elevated roads, bridges and buildings, and the effect is different depending on the concentration of natural radionuclides contained in the construction materials^22,28^. If many materials containing high concentrations of natural radionuclides are utilized, it is expected that the absorbed dose rate will increase. In the present study, lower concentrations of natural radionuclides which were below the worldwide average were observed (Table 1). Thus, the newly constructed built-up environments in those areas where are represented by a metropolitan area such as Ha Noi City (#17; population density, 2013 person/km^2^ in 2011) might have utilized low background building materials, and they might have functioned as shielding materials toward terrestrial gamma-ray radiation. On the other hand, the ratios of the difference observed at Binh Phuoc (#45; population density, 628 person/km^2^ in 2011) and Dong Nai (#46; population density, 268 person/km^2^ in 2011) Provinces were also low (0.5), the same as the urban Ha Noi City, but this was attributed to the shielding effect by water because lakes occupy large parts of those provinces. These areas have not been affected by urbanization and it is expected that the difference in the absorbed ratio in air will not be changed in the future.

The remaining 45% of all data (*n* = 462) were higher (ratio of difference > 1.0) compared to the ratios from soil concentrations. In particular, higher values over 1.5 were observed in the built-up environment of An Giang (#55) and King Giang (#62) Provinces and on Phu Quoc Island (#64). That could be explained by differences of the asphalt cover construction method. In southern Vietnam, crushed granitic stones with diameters of about 10–15 cm have been used under the asphalt cover as a roadbed^17^, and as a result a higher absorbed dose rate in air has been measured on the asphalt compared to the dose rate measured on a bare surface, unlike in northern Vietnam (Supplementary Figure S1). Additionally, activity concentrations of natural radionuclides in soil of those areas had lower values (Fig. 5), and it was expected that those building materials had been brought from another area. In view of balancing building materials under the asphalt cover and original activity concentrations in soil, absorbed dose rates in air increased in those areas. If the survey area is limited to a small area, it might be possible to use the change of absorbed dose rates as an indicator of urbanization for that area by continuing direct measurement of absorbed dose rate.

The H_ex_ clearly changed depending on urbanization, landform, construction methods, types of building materials and original activity concentrations in soil (Fig. 6). The H_ex_ of the built-up environment for mainly urban areas which were densely covered by artificial structures decreased, whereas that on mainly rural areas formed by the low background basement geology had increased H_ex_ compared to the original value which was estimated from soil concentrations. The impact of building materials on the built-up environment related to urbanization can be assessed as ranging from 0.4 to 2.3 times higher H_ex_ with the average H_ex_ value being 0.9 times higher based on the original value. Such future changes can be expected in other developing countries.

**Figure 8:**
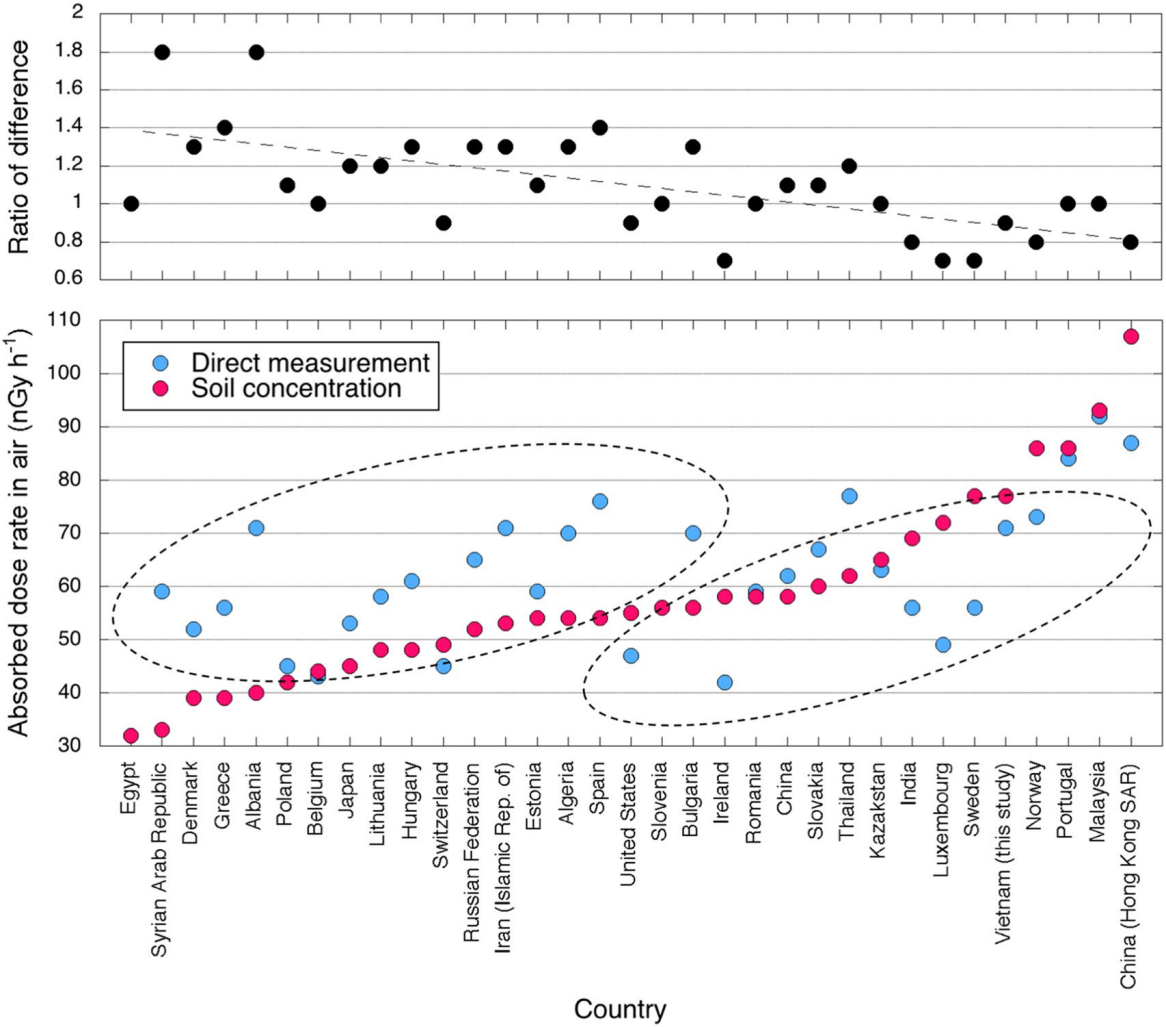
Measured absorbed dose rates in air from direct measurements and from soil concentration, and ratios of differences (direct measurements/soil concentrations) for individual countries.

Changes of absorbed dose rate in air in the built-up environment in individual countries^21^ can be classified broadly into two groups based on absorbed dose rate in air measured from soil concentrations (i.e., activity concentrations in soil) as shown in Fig. 8. If the higher dose rate comes from the soil concentration, building materials have functioned as shielding materials. The different amount of change depends on many factors such as density of artificial structures, architectural styles and contents of natural radionuclides in building materials, Vietnam is classified into this group same as Norway and Sweden, etc. Conversely, if the lower dose rate comes from soil concentrations, the building materials themselves have functioned as sources, and changes of absorbed dose rate in air in the built-up environment will be large. Even when evaluated locally for Ha Noi City (#17) and Phu Quoc Island (#64), this finding is consistent with world trends. Hence, the changes of absorbed dose rate in air in the built-up environment after urban development can be partly predicted by measuring activity concentrations in soil.

The UNSCEAR report^21^ estimated that the average worldwide exposure to natural radiation sources is 2.4 mSv year^−1^. External terrestrial radiation accounts for 0.48 mSv year^−1^. Exposure from natural radiation sources can be categorized into the existing exposure situations presented by ICRP^18^ and IAEA^29^. Additionally, IAEA considered the situation in which the activity concentration of no radionuclide in either the uranium decay chain or the thorium decay chain exceeds 1 kBq kg^−1^ and the activity concentration of ^40^K does not exceed 10 kBq kg^−1^. The reference levels for this exposure situation was set as 1–20 mSv year^−1^^18,29^. In Vietnam the average annual doses calculated from the direct measurements and from the soil concentrations are below this reference level. Therefore, it can be considered that there is no new radiological risk from the built-up environment for Vietnam.

## Methods

### Car-borne survey

The measurements of the count rates from natural radionuclides such as ^40^K, ^238^U series and ^232^Th series elements were carried out using a car-borne survey technique during August and December 2015, July 2016, February and August 2017, March and August 2018 and March 2019 in all of Vietnam (331,212 km^2^). A car-borne survey technique is very useful to make a fast assessment of the dose rate in a large area^30,31^. The survey routes encompassed 58 provinces and five cities (Fig. 1a). Main roads excluding express ways were used to the extent possible, primarily centered on residential areas (Fig. 1b). The total distance of the survey routes was 28,201 km long. The routes travelled over asphalt pavements and measurements used a 3-in. × 3-in. NaI(Tl) scintillation spectrometer with a global positioning system (EMF-211, EMF Japan Co., Osaka, Japan). This spectrometer had been calibrated by an accreditation body according to ISO/IEC 17,025. The NaI(Tl) scintillation spectrometer was positioned 1 m above the ground surface at the center of the car. There were two researchers and a driver in each car during the measurements. Measurement of count rate inside the car was performed every 30 s while the car was moving with a speed around 40 km h^−1^. Latitude and longitude at each measurement point were measured at the same time as the count rates, and the count rates within gamma-ray energies of 50 keV–3.2 MeV were recorded. The contribution of cosmic rays to the gamma-ray pulse height distribution was subtracted using the energy stored in the range from 3.0 to 3.2 MeV^32^. The photon peaks of ^40^K (E_*γ*_ = 1.464 MeV) and ^208^Tl (E_*γ*_ = 2.615 MeV) were used for gamma-ray energy calibration from the channel number and gamma-ray energy before the measurements. The peak position was determined accurately by smoothing the gamma-ray pulse height distribution. Because count rates were measured inside the car, shielding by the car body was also estimated by making measurements inside and outside the care at 30-s intervals during 2 min at 462 locations (red circles in Fig. 1b). Those measurements were done above asphalt surfaces. The shielding factor related to the car body (*SF*_carbody_) was calculated from the correlation between count rates inside and outside the car. In this study, *SF*_carbody_ values were found of 2.01 and 1.71. The correlation between count rates inside and outside the car is shown in Supplementary Figure S2. Additionally, those measurements were done above bare surfaces for 2 min to estimate shielding effect by asphalt pavement (*SF*_aspahlt_) at 319 locations and *SF*_aspahlt_ values were found of 1.37 for southern Vietnam and 0.96 for northern Vietnam. The correlation between dose rates in air and count rates outside the car is given in Supplementary Figure S1. The gamma-ray pulse height distributions were also measured outside the car above bare surfaces for 10 min, at 462 locations (red circles in Fig. 1b) for estimating the dose rate conversion factor (*DCF*) (nGy h^−1^/cps). The gamma-ray pulse height distributions were then unfolded using the 22 × 22 response matrix method^33^ and absorbed dose rates in air^1^ were calculated. These calculated dose rates were used to estimate *DCF* as the correlation between dose rates and count rates outside the car because it is difficult to obtain the photon peaks in the 30-s measurement of the car-borne survey. In this study, *DCF* of 0.14 nGy h^−1^/cps was found. The correlation between count rates on the asphalt surface and absorbed dose rate in air is given in Supplementary Figure S3. The above three factors were multiplied by the corrected count rates outside the car, and the absorbed dose rates (nGy h^−1^) were calculated. Thus, the absorbed dose rate in air outside the car at 1 m above the ground surface (*D*_*air*_) can be calculated using Eq. (1):

1 $${D}_{air}={C}_{in}\times {SF}_{carbody}\times {SF}_{asphalt}\times DCF$$

where *C*_*in*_ is the count rate (cps) inside the car obtained by the measurements for every 30-s interval. All obtained data from the car-borne surveys were plotted on a distribution of absorbed dose rates in air in Vietnam using a minimum curvature algorithm of GMT^34^. This is the method for interpolating data by presuming a smooth curved surface from the data of individual points.

### Annual effective dose

The conversion coefficient from absorbed dose rate in air to effective dose^6,18^ (0.7 Sv G year^−1^) for an adult, and the outdoor (0.2) and indoor (0.8) occupancy factors^21^ were used for estimating the annual outdoor (OAED) and indoor (IAED) annual effective doses based on the absorbed dose rate in air (*D*_air_) measured from the car-borne survey; OAED and IAED (mSv year^−1^) were calculated using Eqs. (2) and (3)^25^.

2 $$OAED={D}_{air}\times 0.7\times 8760\times 0.2\times {10}^{-6}$$

3 $$IAED={D}_{air}\times 0.7\times 8760\times 0.8\times {10}^{-6}$$

### Calculation of activity concentrations of ^238^U, ^232^Th and ^40^K from measured absorbed dose rate in air

The gamma-ray pulse height distributions obtained 1 m above bare surfaces at 462 locations (Fig. 1b) were converted to the energy bin spectrum of incident gamma-rays which is a distribution of gamma-ray flux density to each energy bin to estimate the distribution of activity concentrations of ^40^K, ^238^U series and ^232^Th series for all of Vietnam using the 22 × 22 response matrix method^33^. In this method, energy bins which are unequal intervals in the gamma-ray energy range from 0 to 3.2 MeV were set: 1.464 MeV for ^40^K, 1.765 MeV and 2.205 MeV for ^214^Bi (^238^U series) and 2.615 MeV for ^208^Tl (^232^Th series). The energy intervals for the bins were given from the literature^33^. The calculation for the 22 × 22 response matrix for the 3-in. × 3-in. NaI(Tl) scintillation spectrometer was done using the Monte Carlo code, SPHERIX^35^. The gamma-ray flux density and dose rate per unit solid angle were assumed to be almost isotropic in a natural environment. After unfolding the gamma-ray pulse height distribution, clear peaks from ^40^K (energy range 1.39–1.54 MeV), ^214^Bi (energy range 1.69–1.84 MeV and 2.10–2.31 MeV) and ^208^Tl (energy range 2.51–2.27 MeV) were observed in the spectrum. Additionally, the gamma-ray flux densities per unit activity concentrations of the ^40^K, ^238^U series and ^232^Th series were calculated to evaluate each activity concentration (Bq kg^−1^) of the natural radionuclides using the one-dimensional Monte Carlo gamma transport code^36^.

### Measurement of activity concentration in soils

The soil samples were collected from a layer extending to 15 cm below the ground surface at 462 locations (red circles in Fig. 1b). Samples were dried for 24 h at 110 °C and then sieved. Particles less than 2 mm in size were retained for the activity concentration (Bq kg^−1^) measurement. After homogenizing the sieved samples, the particle samples were packed in a U-8 polypropylene container. Activity concentrations of ^40^K (E_*γ*_ = 1.461 MeV), ^214^Pb (E_*γ*_ = 0.351 MeV) and ^214^Bi (E_*γ*_ = 0.609 MeV) for ^238^U series and ^228^Ac (E_*γ*_ = 911 MeV) for ^232^Th series in each sample were measured for 30,000 s with a high-purity germanium semiconductor detector (GMX10P, ORTEC, Oak Ridge, TN). The standard volume source (U8 type; 5, 10, 20, 30, or 50 mm height) which had been calibrated by an accreditation body according to ISO/IEC 17025 was for estimating counting efficiency determination. This source contained nine radionuclides (Mn-54, Cr-51, Co-57, Co-60, Sr-85, Y-88, Cd-109, Cs-137, Ce-139). The relative efficiency of this detector was 17.50%, and activity concentrations were calculated by the absolute method. The activity concentration of ^238^U series was calculated using a weighted average of the activity concentrations of ^214^Pb and ^214^Bi^37^.

### Absorbed dose rate estimated from activity concentrations in soil

There is a direct connection between activity concentrations in soil and terrestrial gamma-ray radiation. The absorbed dose rate in air 1 m above the ground surface can be calculated based on the measured activity concentrations of ^238^U, ^232^Th and ^40^K using Eq. (4)^38^:

4 $${D}_{soil}=0.43{A}_{U}+0.666{A}_{Th}+0.042{A}_{K}$$

where *A*_*U*_, *A*_*Th*_ and *A*_*K*_ are the concentrations of ^238^U, ^232^Th and ^40^K in Bq kg^−1^, respectively.

### Radium equivalent activity (***Ra***_***eq***_)

Radium equivalent index in Bq kg^−1^ is a widely utilized radiological hazard index. It is a convenient index to compare the specific activities of samples containing different amounts of ^238^U, ^232^Th and ^40^K^39^.

5 $${Ra}_{eq}={A}_{U}+1.43{A}_{Th}+0.077{A}_{K}$$

It has been assumed that 370 Bq kg^−1^ of ^238^U or 259 Bq kg^−1^ of ^232^Th or 4,810 Bq kg^−1^ of ^40^K produce the same gamma dose rate and that gamma dose rate is related to the external gamma dose and internal dose due to radon and its daughters such as thoron.

### Radiological hazard index (***H***_ex_)

The external hazard index due to gamma radiation was calculated using Eq. (6)^9,25^:

6 $${H}_{ex}=\frac{{A}_{U}}{370}+\frac{{A}_{Th}}{259}+\frac{{A}_{K}}{4810}\le 1$$

where *A*_*U*_, *A*_*Th*_ and *A*_*K*_ are the concentrations in Bq kg^−1^ of ^238^U, ^232^Th and ^40^K, respectively. When the external hazard index values did not exceed the acceptable limit which is less than unity (H_ex_ < 1), radiation hazards could be assumed to be negligible in the study area.

## Supplementary information

Supplementary information
