# Supplementary material for: Distribution of gamma radiation dose rate related with natural radionuclides in all of Vietnam and radiological risk assessment of the built-up environment
Source: Sci Rep. 2020 Jul 24;10:12428. doi: 10.1038/s41598-020-69003-0 (PMC7381640; doi:10.1038/s41598-020-69003-0)
Supplement: Supplementary file 1 — Supplementary information [file 41598_2020_69003_MOESM1_ESM.docx]

**Supplementary information**

**Distribution of gamma radiation dose rate related with natural radionuclides in all of Vietnam and radiological risk assessment of the built-up environment**

Kazumasa Inoue^1,*^, Masahiro Fukushi^1^, Tan Van Le^1,2^, Hiroshi Tsuruoka^1,3^,

Shogo Kasahara^1^, Veerasamy Nimelan^1^

^1^ Department of Radiological Sciences, Graduate School of Human Health Sciences, Tokyo Metropolitan University, Tokyo, 116-8551, Japan

^2^ Department of Radiology, Cho Ray Hospital, Ho Chi Minh City, 72713, Vietnam

^3^ Department of Radiological Sciences, Tsukuba International University, Ibaraki, 300-0051, Japan

* kzminoue@tmu.ac.jp

**
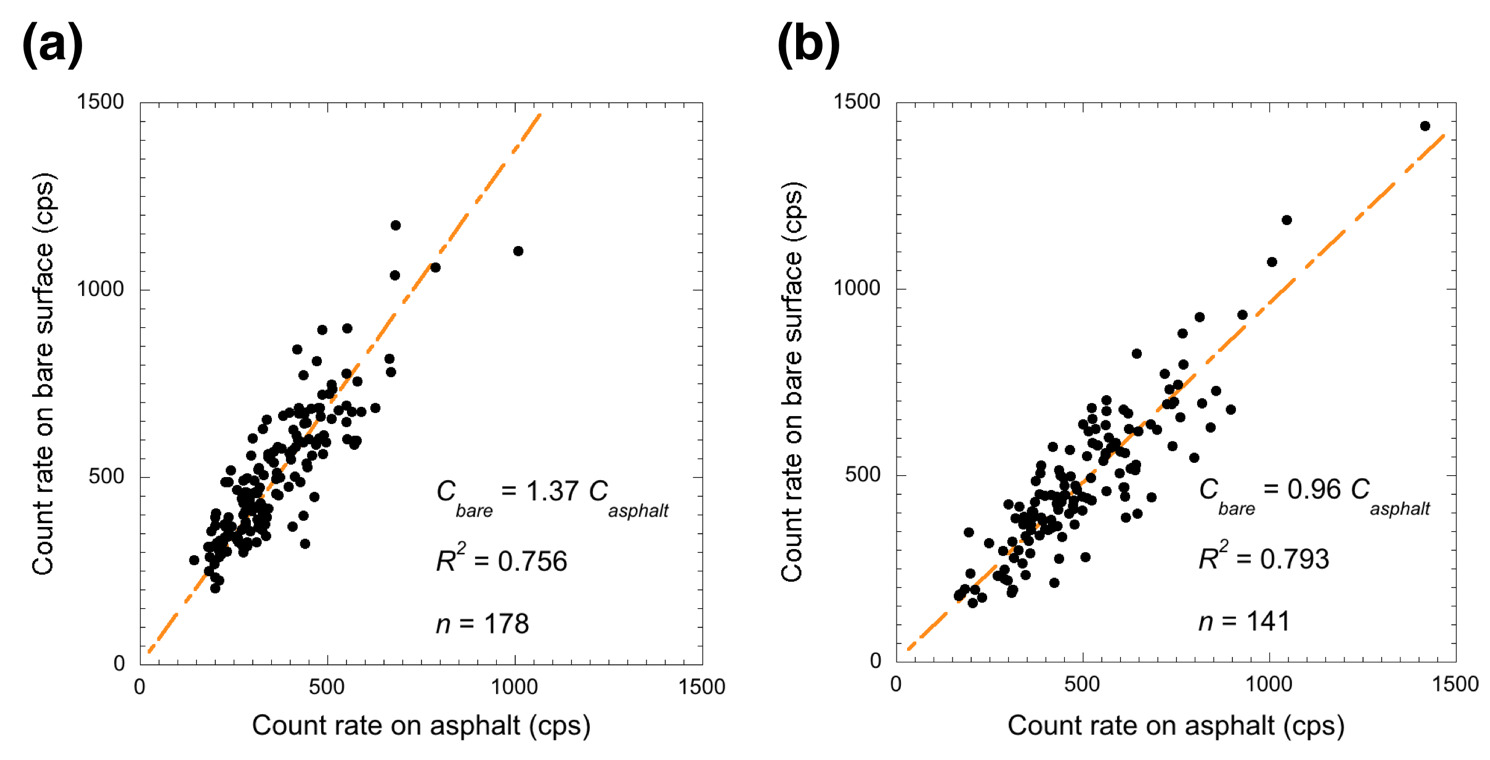
**

**Figure S1.** Correlations between count rates on asphalt and bare surface for (a) southern Vietnam and for (b) northern Vietnam. The slopes of these regression lines were used as the shielding factors by asphalt pavement, 1.37 for southern Vietnam and 0.96 for northern Vietnam.

**
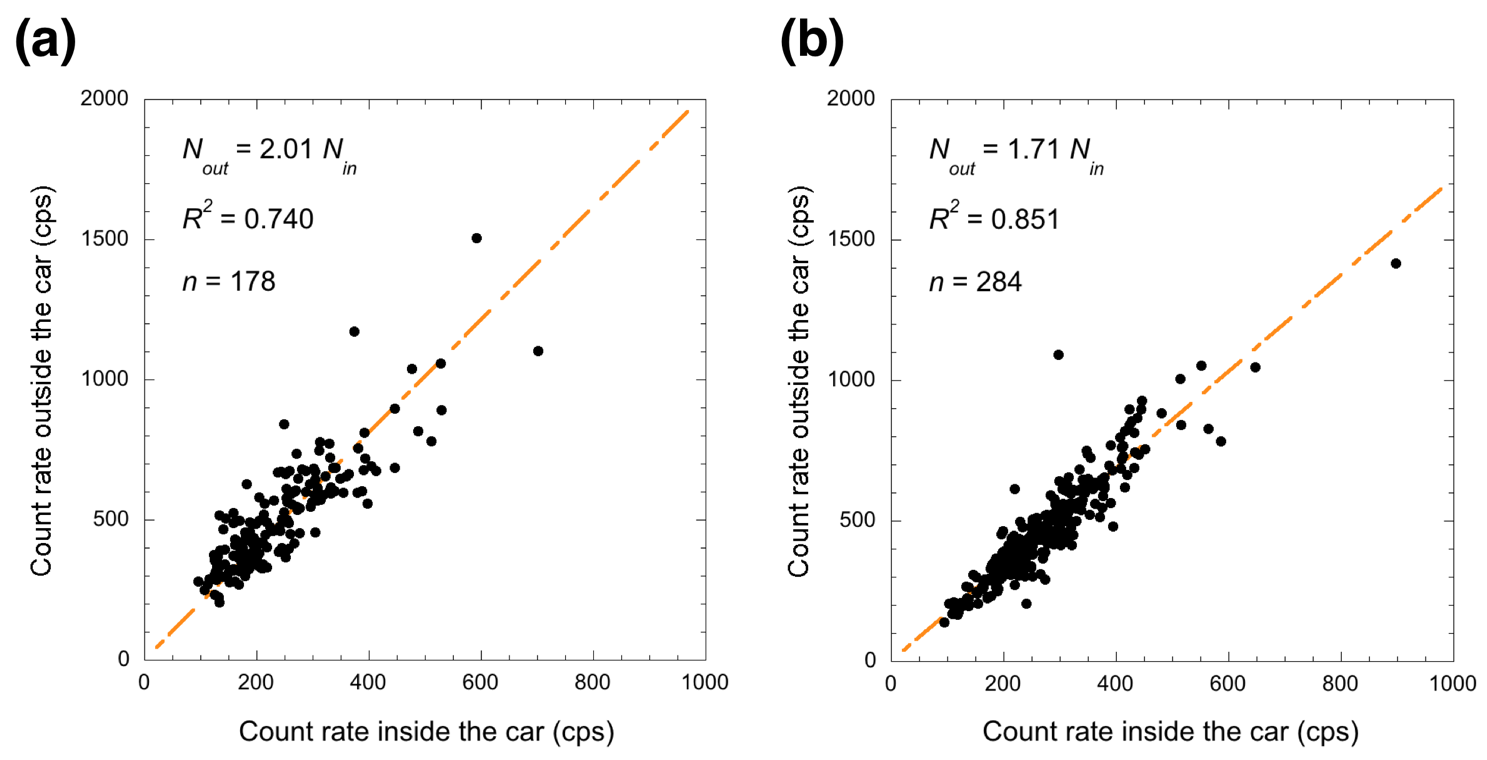
**

**Figure S2.** Correlations between count rates inside and outside the cars. The slopes of these regression lines were used as the shielding factors by the car body, (a) 2.01 for Car #1 and (b) 1.71 for Car #2.

**
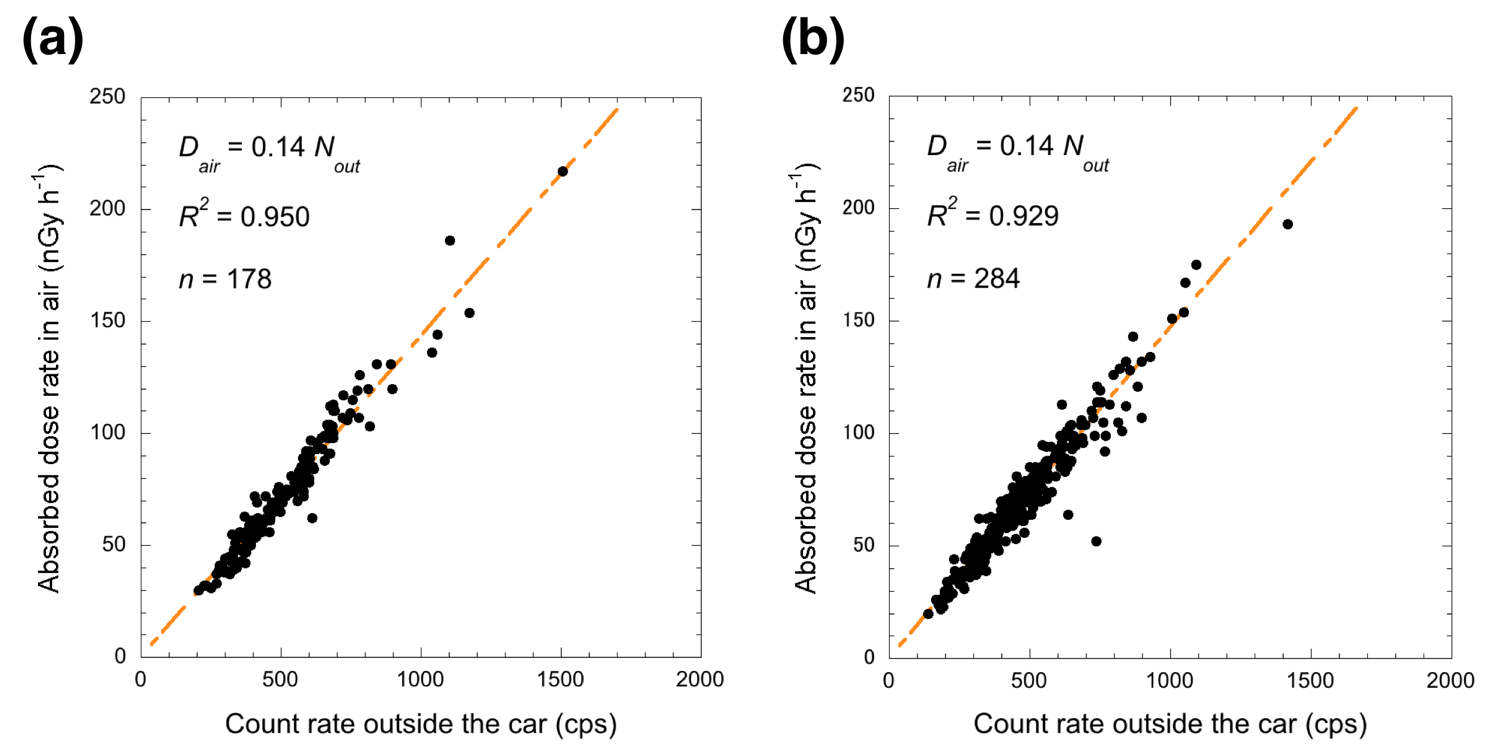
**

**Figure S3.** Correlations between absorbed dose rates in air and count rates outside the cars. The slopes of these regression lines were used as the dose conversion factor, 0.14 nGy h^-1^/cps for (a) Car #1 and (b) Car #2.

**Table S1.** Activity concentrations calculated from measurements of gamma-ray pulse height distributions.

| No.^a^ | Municipality | *n* | Activity concentration (Bq kg^-1^) | | | | | |
| --- | --- | --- | --- | --- | --- | --- | --- | --- |
|  |  |  | ^238^U | | ^232^Th | | ^40^K | |
|  |  |  | Average | Range | Average | Range | Average | Range |
| 1 | Lai Chau | 6 | 174±124 | 57-395 | 42±16 | 26-66 | 473±70 | 409-601 |
| 2 | Dien Bien | 3 | 93±39 | 49-120 | 44±12 | 36-58 | 466±75 | 401-548 |
| 3 | Lao Cai | 6 | 62±24 | 39-90 | 57±27 | 25-91 | 431±102 | 236-518 |
| 4 | Ha Giang | 10 | 84±61 | 41-233 | 46±21 | 21-92 | 564±309 | 237-1288 |
| 5 | Cao Bang | 6 | 45±20 | 30-84 | 38±19 | 19-72 | 347±295 | 85-884 |
| 6 | Tuyen Quang | 7 | 81±19 | 53-113 | 44±21 | 17-77 | 376±237 | 94-795 |
| 7 | Yen Bai | 5 | 59±29 | 24-101 | 44±25 | 12-71 | 559±434 | 266-1319 |
| 8 | Bac Kan | 7 | 44±6 | 33-50 | 51±18 | 25-79 | 483±137 | 248-698 |
| 9 | Lang Son | 10 | 53±18 | 28-82 | 60±23 | 29-101 | 437±267 | 98-889 |
| 10 | Thai Nguyen | 6 | 42±13 | 26-62 | 36±15 | 23-62 | 310±262 | 149-818 |
| 11 | Phu Tho | 12 | 102±59 | 40-198 | 37±9 | 25-48 | 302±144 | 30-494 |
| 12 | Vinh Phuc | 3 | 74±5 | 70-79 | 58±10 | 52-70 | 428±65 | 357-483 |
| 13 | Bac Giang | 8 | 31±8 | 21-41 | 33±6 | 23-44 | 218±83 | 88-347 |
| 14 | Quang Ninh | 8 | 56±20 | 29-94 | 62±33 | 29-130 | 386±90 | 287-547 |
| 15 | Bac Ninh | 2 | 29±2 | 28-31 | 28±3 | 25-30 | 268±59 | 226-309 |
| 16 | Son La | 6 | 48±18 | 26-78 | 33±10 | 19-46 | 380±93 | 274-484 |
| 17 | Ha Noi | 3 | 47±11 | 34-54 | 23±11 | 12-34 | 280±111 | 153-357 |
| 18 | Hai Duong | 3 | 29±1 | 27-30 | 27±4 | 23-32 | 300±47 | 272-355 |
| 19 | Hoa Binh | 5 | 54±11 | 40-70 | 37±12 | 25-52 | 383±246 | 128-730 |
| 20 | Hai Phong | 3 | 52±28 | 32-72 | 32±4 | 29-35 | 372±58 | 331-413 |
| 21 | Hung Yen | 3 | 39±9 | 29-44 | 28±4 | 24-32 | 412±92 | 320-505 |
| 22 | Ha Nam | 3 | 43±4 | 40-47 | 16±12 | 9-30 | 312±120 | 198-438 |
| 23 | Thai Binh | 3 | 54±2 | 53-56 | 27±2 | 26-29 | 393±25 | 374-422 |
| 24 | Ninh Binh | 3 | 44±1 | 43-46 | 27±5 | 22-32 | 336±15 | 318-347 |
| 25 | Nam Dinh | 3 | 37±4 | 33-40 | 24±17 | 12-44 | 343±152 | 221-513 |
| 26 | Thanh Hoa | 6 | 46±19 | 26-75 | 43±22 | 15-66 | 474±257 | 144-784 |
| 27 | Nghe An | 14 | 50±21 | 29-112 | 52±24 | 31-120 | 488±179 | 221-869 |
| 28 | Ha Tinh | 4 | 52±19 | 34-79 | 52±11 | 40-68 | 574±161 | 342-700 |
| 29 | Quang Binh | 9 | 51±22 | 20-98 | 52±18 | 25-85 | 371±216 | 104-670 |

*Continued*

| No.^a^ | Municipality | *n* | Activity concentration (Bq kg^-1^) | | | | | |
| --- | --- | --- | --- | --- | --- | --- | --- | --- |
|  |  |  | ^238^U | | ^232^Th | | ^40^K | |
|  |  |  | Average | Range | Average | Range | Average | Range |
| 30 | Quang Tri | 7 | 41±12 | 23-57 | 53±17 | 36-75 | 544±200 | 311-937 |
| 31 | Thua Thien-Hue | 5 | 62±33 | 35-117 | 46±12 | 32-63 | 646±184 | 498-960 |
| 32 | Da Nang | 8 | 54±7 | 46-65 | 70±11 | 57-92 | 737±65 | 626-816 |
| 33 | Quang Nam | 14 | 60±32 | 29-106 | 52±19 | 26-82 | 616±212 | 246-1038 |
| 34 | Quang Ngai | 11 | 80±34 | 41-155 | 77±37 | 28-147 | 745±245 | 354-1117 |
| 35 | Binh Dinh | 11 | 67±16 | 43-87 | 75±20 | 48-104 | 923±133 | 723-1179 |
| 36 | Phu Yen | 8 | 44±10 | 30-57 | 42±15 | 27-65 | 719±209 | 505-1074 |
| 37 | Khanh Hoa | 19 | 50±18 | 18-104 | 43±13 | 11-70 | 540±146 | 234-874 |
| 38 | Ninh Thuan | 20 | 49±13 | 34-92 | 48±14 | 28-82 | 714±156 | 408-947 |
| 39 | Binh Thuan | 18 | 48±19 | 19-93 | 43±19 | 23-95 | 440±142 | 144-659 |
| 40 | Kon Tum | 7 | 49±14 | 28-71 | 51±18 | 24-80 | 527±141 | 307-702 |
| 41 | Gia Lai | 11 | 32±12 | 19-56 | 38±16 | 17-65 | 452±204 | 155-851 |
| 42 | Dac Lac | 11 | 36±11 | 22-55 | 37±15 | 23-71 | 515±164 | 182-729 |
| 43 | Dac Nong | 9 | 25±11 | 12-43 | 21±10 | 12-43 | 283±151 | 116-564 |
| 44 | Lam Dong | 14 | 53±29 | 26-136 | 50±36 | 23-157 | 541±202 | 192-893 |
| 45 | Binh Phuoc | 4 | 22±10 | 15-37 | 19±12 | 11-37 | 281±246 | 91-637 |
| 46 | Dong Nai | 15 | 38±13 | 14-69 | 33±7 | 21-45 | 471±154 | 174-772 |
| 47 | Ba Ria-Vung Tau | 7 | 28±7 | 22-42 | 29±4 | 25-36 | 404±145 | 257-674 |
| 48 | Tay Ninh | 20 | 38±11 | 27-68 | 34±8 | 22-50 | 281±161 | 56-685 |
| 49 | Binh Duong | 11 | 35±8 | 25-51 | 36±7 | 28-46 | 345±121 | 159-522 |
| 50 | Ho Chi Minh | 11 | 33±8 | 24-50 | 34±6 | 23-44 | 412±149 | 97-703 |
| 51 | Long An | 4 | 45±4 | 40-48 | 47±7 | 39-55 | 619±138 | 448-784 |
| 52 | Tien Giang | 5 | 30±5 | 24-35 | 30±8 | 22-43 | 422±89 | 355-571 |
| 53 | Ben Tre | 4 | 37±6 | 32-45 | 37±7 | 32-48 | 466±103 | 350-601 |
| 54 | Dong Thap | 7 | 33±8 | 25-44 | 34±8 | 25-47 | 530±106 | 372-683 |
| 55 | An Giang | 3 | 53±5 | 49-59 | 44±9 | 34-53 | 942±160 | 841-1126 |
| 56 | Vinh Long | 3 | 30±8 | 25-38 | 32±7 | 26-42 | 560±112 | 462-683 |
| 57 | Can Tho | 2 | 27±7 | 22-32 | 21±12 | 13-29 | 476±300 | 264-688 |
| 58 | Hau Giang | 1 | 35 | - | 43 | - | 665 | - |
| 59 | Tra Vinh | 2 | 30±1 | 29-30 | 32±6 | 28-37 | 460±41 | 431-488 |
| 60 | Soc Trang | 3 | 39±10 | 28-47 | 38±14 | 29-54 | 602±96 | 515-705 |
| 61 | Bac Lieu | 2 | 38±1 | 37-38 | 40±9 | 34-46 | 664±46 | 631-696 |

*Continued*

| No.^a^ | Municipality | *n* | Activity concentration (Bq kg^-1^) | | | | | |
| --- | --- | --- | --- | --- | --- | --- | --- | --- |
|  |  |  | ^238^U | | ^232^Th | | ^40^K | |
|  |  |  | Average | Range | Average | Range | Average | Range |
| 62 | Kien Giang | 7 | 47±12 | 31-66 | 42±11 | 29-64 | 723±165 | 432-961 |
| 63 | Ca Mau | 4 | 42±13 | 28-56 | 35±6 | 27-40 | 601±181 | 459-866 |
| 64 | Phu Quoc | 10 | 33±14 | 19-65 | 26±12 | 14-56 | 366±260 | 132-844 |

^a^ The numbers refer to the designations in Figure 1a.

**Table S2.** Activity concentrations of natural radionuclides in soil.

| No.^a^ | Municipality | *n* | Activity concentration (Bq kg^-1^) | | | | | |
| --- | --- | --- | --- | --- | --- | --- | --- | --- |
|  |  |  | ^238^U | | ^232^Th | | ^40^K | |
|  |  |  | Average | Range | Average | Range | Average | Range |
| 1 | Lai Chau | 6 | 70±42 | 35-149 | 79±32 | 27-126 | 747±234 | 459-1014 |
| 2 | Dien Bien | 3 | 49±1 | 48-51 | 91±11 | 80-103 | 890±46 | 838-928 |
| 3 | Lao Cai | 6 | 54±24 | 31-100 | 93±40 | 30-124 | 550±133 | 356-758 |
| 4 | Ha Giang | 10 | 60±44 | 23-146 | 84±31 | 28-124 | 823±317 | 487-1270 |
| 5 | Cao Bang | 6 | 25±9 | 11-37 | 46±22 | 17-81 | 338±115 | 218-581 |
| 6 | Tuyen Quang | 7 | 45±25 | 23-98 | 90±42 | 41-168 | 604±299 | 163-1017 |
| 7 | Yen Bai | 5 | 45±25 | 11-77 | 72±54 | 5-140 | 644±375 | 382-1290 |
| 8 | Bac Kan | 7 | 38±14 | 15-59 | 71±25 | 34-119 | 574±155 | 326-834 |
| 9 | Lang Son | 10 | 39±14 | 7-52 | 76±30 | 14-112 | 671±326 | 259-1320 |
| 10 | Thai Nguyen | 6 | 29±16 | 14-61 | 41±36 | 11-111 | 265±81 | 169-343 |
| 11 | Phu Tho | 12 | 36±13 | 19-56 | 70±34 | 28-141 | 475±190 | 303-835 |
| 12 | Vinh Phuc | 3 | 56±15 | 47-73 | 106±21 | 85-127 | 662±54 | 600-696 |
| 13 | Bac Giang | 8 | 24±9 | 12-39 | 37±12 | 20-55 | 344±74 | 267-491 |
| 14 | Quang Ninh | 8 | 31±14 | 19-63 | 61±36 | 23-125 | 497±215 | 234-916 |
| 15 | Bac Ninh | 2 | 20±4 | 19-21 | 31±8 | 29-33 | 376±32 | 326-386 |
| 16 | Son La | 6 | 53±35 | 27-111 | 61±13 | 50-83 | 658±206 | 313-942 |
| 17 | Ha Noi | 3 | 44±19 | 22-56 | 56±22 | 35-79 | 505±104 | 395-602 |
| 18 | Hai Duong | 3 | 26±7 | 19-33 | 41±9 | 31-50 | 329±1 | 329-330 |
| 19 | Hoa Binh | 5 | 49±34 | 24-109 | 47±13 | 32-66 | 492±218 | 259-801 |
| 20 | Hai Phong | 3 | 27±8 | 18-33 | 48±1 | 47-49 | 663±44 | 593-680 |
| 21 | Hung Yen | 3 | 24±13 | 11-36 | 44±30 | 10-64 | 455±120 | 317-532 |
| 22 | Ha Nam | 3 | 33±15 | 22-49 | 47±21 | 26-68 | 632±198 | 479-856 |
| 23 | Thai Binh | 3 | 35±7 | 30-42 | 58±7 | 50-64 | 703±83 | 619-786 |
| 24 | Ninh Binh | 3 | 48±14 | 38-63 | 63±34 | 24-88 | 519±262 | 237-754 |
| 25 | Nam Dinh | 3 | 29±7 | 22-36 | 32±17 | 16-50 | 414±194 | 210-595 |
| 26 | Thanh Hoa | 6 | 22±9 | 6-34 | 44±21 | 13-74 | 540±302 | 23-890 |
| 27 | Nghe An | 14 | 36±21 | 8-79 | 62±28 | 24-122 | 544±254 | 174-1114 |
| 28 | Ha Tinh | 4 | 22±8 | 13-34 | 42±14 | 28-64 | 474±241 | 252-819 |
| 29 | Quang Binh | 9 | 36±35 | 7-124 | 60±37 | 22-140 | 387±231 | 158-796 |

*Continued*

| No.^a^ | Municipality | *n* | Activity concentration (Bq kg^-1^) | | | | | |
| --- | --- | --- | --- | --- | --- | --- | --- | --- |
|  |  |  | ^238^U | | ^232^Th | | ^40^K | |
|  |  |  | Average | Range | Average | Range | Average | Range |
| 30 | Quang Tri | 7 | 27±13 | 8-44 | 61±36 | 6-106 | 617±228 | 221-872 |
| 31 | Thua Thien-Hue | 5 | 48±32 | 10-102 | 67±46 | 2-137 | 635±340 | 220-1210 |
| 32 | Da Nang | 8 | 32±21 | 10-72 | 62±49 | 16-157 | 631±237 | 366-1060 |
| 33 | Quang Nam | 14 | 35±12 | 18-54 | 62±22 | 29-97 | 555±328 | 120-1340 |
| 34 | Quang Ngai | 11 | 39±15 | 25-73 | 75±37 | 33-160 | 541±184 | 313-875 |
| 35 | Binh Dinh | 11 | 44±24 | 15-102 | 79±41 | 19-133 | 1036±543 | 327-1910 |
| 36 | Phu Yen | 8 | 38±18 | 17-68 | 73±39 | 30-125 | 1038±366 | 673-1687 |
| 37 | Khanh Hoa | 19 | 46±21 | 18-91 | 66±25 | 24-97 | 777±263 | 543-1425 |
| 38 | Ninh Thuan | 20 | 34±14 | 8-58 | 60±38 | 1-142 | 885±447 | 4-1480 |
| 39 | Binh Thuan | 18 | 32±15 | 14-54 | 46±23 | 0-74 | 606±337 | 207-1200 |
| 40 | Kon Tum | 7 | 40±18 | 23-70 | 99±35 | 59-149 | 515±526 | 86-1650 |
| 41 | Gia Lai | 11 | 42±41 | 10-160 | 78±48 | 28-173 | 643±468 | 88-1850 |
| 42 | Dac Lac | 11 | 26±14 | 9-55 | 47±35 | 0-115 | 515±435 | 122-1260 |
| 43 | Dac Nong | 9 | 20±8 | 6-32 | 41±15 | 15-60 | 268±219 | 36-694 |
| 44 | Lam Dong | 14 | 35±17 | 12-66 | 64±32 | 21-126 | 506±333 | 37-1312 |
| 45 | Binh Phuoc | 4 | 30±21 | 14-61 | 55±41 | 17-112 | 369±333 | 56-783 |
| 46 | Dong Nai | 15 | 44±74 | 3-307 | 84±141 | 15-592 | 606±712 | 212-3145 |
| 47 | Ba Ria-Vung Tau | 7 | 22±9 | 12-38 | 28±22 | 0-55 | 364±84 | 239-477 |
| 48 | Tay Ninh | 20 | 27±7 | 11-41 | 35±14 | 1-58 | 266±83 | 56-404 |
| 49 | Binh Duong | 11 | 29±18 | 15-50 | 48±25 | 24-88 | 247±298 | 5-944 |
| 50 | Ho Chi Minh | 11 | 26±8 | 15-37 | 40±16 | 19-69 | 383±176 | 66-653 |
| 51 | Long An | 4 | 45±8 | 34-54 | 59±20 | 43-94 | 583±393 | 179-1144 |
| 52 | Tien Giang | 5 | 21±3 | 19-24 | 30±6 | 26-37 | 466±77 | 399-550 |
| 53 | Ben Tre | 4 | 27±3 | 24-32 | 39±7 | 31-48 | 565±106 | 472-667 |
| 54 | Dong Thap | 7 | 40±40 | 18-112 | 48±31 | 27-101 | 625±259 | 393-921 |
| 55 | An Giang | 3 | 23±7 | 16-31 | 37±14 | 23-51 | 630±271 | 393-932 |
| 56 | Vinh Long | 3 | 57±21 | 36-77 | 75±29 | 50-107 | 1009±424 | 541-1366 |
| 57 | Can Tho | 2 | 44±26 | 25-62 | 72±42 | 42-101 | 898±634 | 450-1346 |
| 58 | Hau Giang | 1 | 33 | − | 54 | − | 791 | − |
| 59 | Tra Vinh | 2 | 27±7 | 20-34 | 51±8 | 43-59 | 788±317 | 426-1014 |
| 60 | Soc Trang | 3 | 35±19 | 18-55 | 56±37 | 28-98 | 528±221 | 363-779 |
| 61 | Bac Lieu | 2 | 24±3 | 22-26 | 36±5 | 32-39 | 408±91 | 344-472 |

*Continued*

| No.^a^ | Municipality | *n* | Activity concentration (Bq kg^-1^) | | | | | |
| --- | --- | --- | --- | --- | --- | --- | --- | --- |
|  |  |  | ^238^U | | ^232^Th | | ^40^K | |
|  |  |  | Average | Range | Average | Range | Average | Range |
| 62 | Kien Giang | 7 | 20±18 | 4-75 | 31±29 | 3-111 | 305±400 | 19-1476 |
| 63 | Ca Mau | 4 | 28±13 | 18-277 | 68±38 | 31-107 | 810±455 | 422-1310 |
| 64 | Phu Quoc | 10 | 5±7 | 0-20 | 7±8 | 11-32 | 40±54 | 15-180 |

^a^ The numbers refer to the designations in Figure 1a.
